# EGFR, HER2, and HER3 protein expression in paired primary tumor and lymph node metastasis of colorectal cancer

**DOI:** 10.1038/s41598-022-17210-2

**Published:** 2022-07-28

**Authors:** Peng Ye, Fanghua Li, Yuanyuan Wei, Yihao Zhang, Jianing Cui, Rui Dai, Hao Chen, Jing Xie, Peiling Cai

**Affiliations:** 1 Department of Anatomy and Histology, School of Preclinical Medicine, Chengdu University, Chengdu, Sichuan Province People’s Republic of China; 2 Department of Pathology, Sichuan Academy of Medical Sciences & Sichuan Provincial People’s Hospital, Chengdu, Sichuan Province People’s Republic of China; 3 Department of Physiology, School of Preclinical Medicine, Chengdu University, Chengdu, Sichuan Province People’s Republic of China; 4 School of Preclinical Medicine, Chengdu University, Chengdu, Sichuan Province People’s Republic of China; 5 Department of Pathology and Clinical Laboratory, Sichuan Provincial Fourth People’s Hospital, Chengdu, Sichuan Province People’s Republic of China

**Keywords:** Targeted therapies, Tumour biomarkers, Tumour heterogeneity

## Abstract

Due to the difficulty in sampling of metastatic tumors, patient selection is commonly based on results of primary tumor samples when metastatic samples are not available. However, due to tumor heterogeneity, metastatic tumors may be different from primary tumors in their phenotypes. The aim of this study was to investigate the expression of EGFR, HER2, and HER3 between primary and lymph node metastatic lesions of colorectal cancer. Paired primary tumors and lymph node metastases from 79 patients with colorectal cancer were retrospectively collected and analyzed for EGFR, HER2, and HER3 expression. High EGFR, HER2, and HER3 expression (2+ and 3+) was found in 64.2%, 66.0%, and 85.0% of primary tumors, and 56.8%, 46.0%, and 76.0% of lymph node metastases, respectively. Correlation rates between primary and metastatic lesions were 67.1%, 63.3%, and 74.7% for EGFR, HER2, and HER3, respectively. Stage IV tumors (with distant metastasis) had higher correlation rates of HER2 expression compared to stage III tumors (without distant metastasis) (*P* = 0.050). Moderate correlation rates in EGFR, HER2, and HER3 expression were observed between primary and metastatic lesions of colorectal cancer. Tumor stage or existence of distant metastasis could serve as potential predictive markers for the correlation of HER2 expression between primary tumors and lymph node metastases of colorectal cancer.

## Introduction

Colorectal cancer (CRC) is the 3rd leading cause of cancer-related death worldwide, which took nearly one million lives in 2018^1^. Tumor metastasis is the major cause of patient death in CRC. As reported in previous studies, 20–25% of the CRC patients showed metastasis when they were firstly diagnosed, and almost half of the patients developed metastasis after progression of the disease^2,3^. The 5-year survival rate of patients with metastatic CRC (mCRC) was only 13.3% which is much lower than that of localized CRC (73.1%). If left untreated, the median survival period of mCRC was only eight months^3,4^.

The human epidermal growth factor receptor (HER) family consists of four members: epithelial growth factor receptor (EGFR), HER2, HER3, and HER4. All the HER family members are receptor tyrosine kinases which consist of extracellular domains, transmembrane segments, and endoplasmic tyrosine kinase domains^5^. The HER family members are activated through formation of homodimers or heterodimers and subsequent phosphorylation of the endoplasmic tyrosine kinase domains^6^. The roles of HER family members in cancer have been well established. EGFR expression is widely up-regulated among different types of epithelial tumors, and therapies targeting EGFR have been used to treat mCRC for nearly two decades^7^. Similar to EGFR, overexpression of HER2 is commonly seen in different types of tumors^6^. Therapies targeting HER2 have been approved for the treatment of HER2-positive breast cancer and HER2-positive gastric cancer^8,9^, and are currently under investigation in preclinical and clinical studies for the treatment of mCRC^10^. HER3 activation is thought to be involved in the resistance mechanism of anti-EGFR and anti-HER2 therapies^11^. In addition, high HER3 expression (immunohistochemistry score 2+ and 3+) was found in 80% of primary lesions and 81% of lymph node metastases of CRC^12^, and therapy directly targeting HER3 is currently under investigation in phase 2 clinical trial (NCT04479436). Unlike the other HER family members, HER4 is not commonly overexpressed in tumors, and HER4 signaling promotes cell differentiation and apoptosis^13^.

Previous studies have found significant genetic diversity between and within tumors, namely tumor heterogeneity, which is a key challenge in personalized cancer medicine^14,15^. Due to the difficulty in tumor sampling, metastatic lesion samples are commonly unavailable, and patient selection in clinical trials or practices is more based on results from primary lesions. However, this may be potentially harmful considering the existence of heterogeneity between primary and metastatic lesions. Several previous studies have compared the expression of EGFR, HER2, or HER3 between primary and distant metastatic lesions of CRC, and the correlation rates ranged from 46.5 to 94.7% for EGFR (46.5%^16^, 94.7%^17^, and 60%^18^), and from 55 to 72.7% for HER2 (55%^18^, 72.7%^19^, and 70.2%^20^).

Lymph node metastasis is the most important prognostic indicator for solid cancer patients, and the distal metastasis of tumor is mostly via lymphatic system^21^. Although not directly related to drug responsiveness of distant metastatic tumors, the genomic profiles of lymph node metastatic tumors may help understand the evolution of tumor cells during the process of metastasis. Some previous studies compared the EGFR, HER2, or HER3 expressions between primary tumor and lymph node metastases of CRC. Shan^19^ found a correlation rate of 89.9% (62/69) in HER2 status between primary tumor and lymph node metastasis of CRC. Wei^22^ compared the immunohistochemistry scores of EGFR, HER2, and HER3 between primary CRC and lymph node metastases, and found moderate to high correlation (EGFR: 69.6%; HER2: 96.4%; HER3: 83.7%). In another study, correlation of low/high HER3 expression between paired primary CRC tumor and lymph node metastases was found in 84 out of 102 (82.4%) patients^23^. However, it is still difficult to draw a conclusion from these results, considering the limited number of these studies. More investigation is required to provide more evidence. In the present study, we further investigated the protein expression of EGFR, HER2, and HER3 in primary lesions and lymph node metastasis of CRC, which could hopefully shed more light on this problem.

## Results

### Patient clinicopathologic characteristics

Paired primary and lymph node metastatic lesion samples were collected from 107 patients. After quality control, samples from 28 patients were excluded due to lack of tumor cells on slides or incomplete patient information. The median age of the patients was 67 years (range 34–87 years), with 38 (48.1%) males and 41 (51.9%) females. The clinicopathologic characteristics of the 79 patients are summarized in Table 1.

**Table 1:** Clinicopathologic characteristics.

Characteristics	n (%) of patients
**Tumor type**	
Typical adenocarcinoma	72 (91.1)
Mucinous adenocarcinoma	7 (8.9)
**Primary tumor location**	
Right colon	13 (16.5)
Left colon	17 (21.5)
Rectum	49 (62.0)
**Differentiation**	
Low	14 (17.7)
Moderate	59 (74.7)
High	6 (7.6)
**Tumor stage**	
III	58 (73.4)
IV	21 (26.6)
**T stage**	
T1 and T2	6 (7.6)
T3 and T4	73 (92.4)
**N stage**	
N1	41 (51.9)
N2	35 (44.3)
N3	3 (3.8)
**M stage**	
M0	58 (73.4)
M1	21 (26.6)

A TNM classification system^57^ was used to define the tumor stage, T stage, N stage and M stage of the tumors.

### Protein overexpression of EGFR, HER2, and HER3

**Figure 1:**
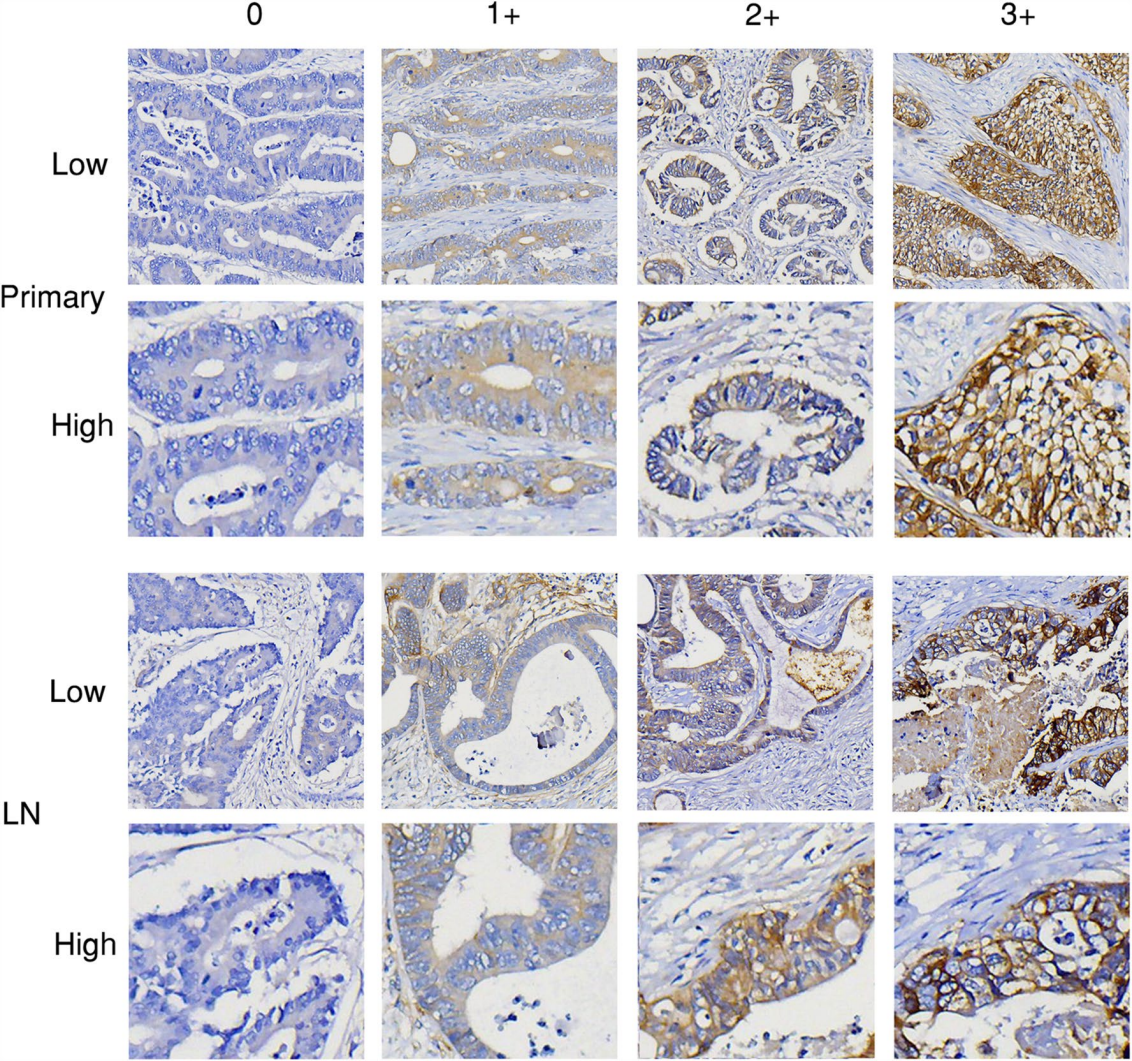
Representative images (EGFR immunohistochemistry staining). Representative images of immunohistochemistry staining scores (0, 1+, 2+, and 3+) for EGFR in primary tumor (Primary) and lymph node metastasis (LN) of CRC. Low: low magnificent power field (× 40); High: high magnificent power field (× 200).

**Figure 2:**
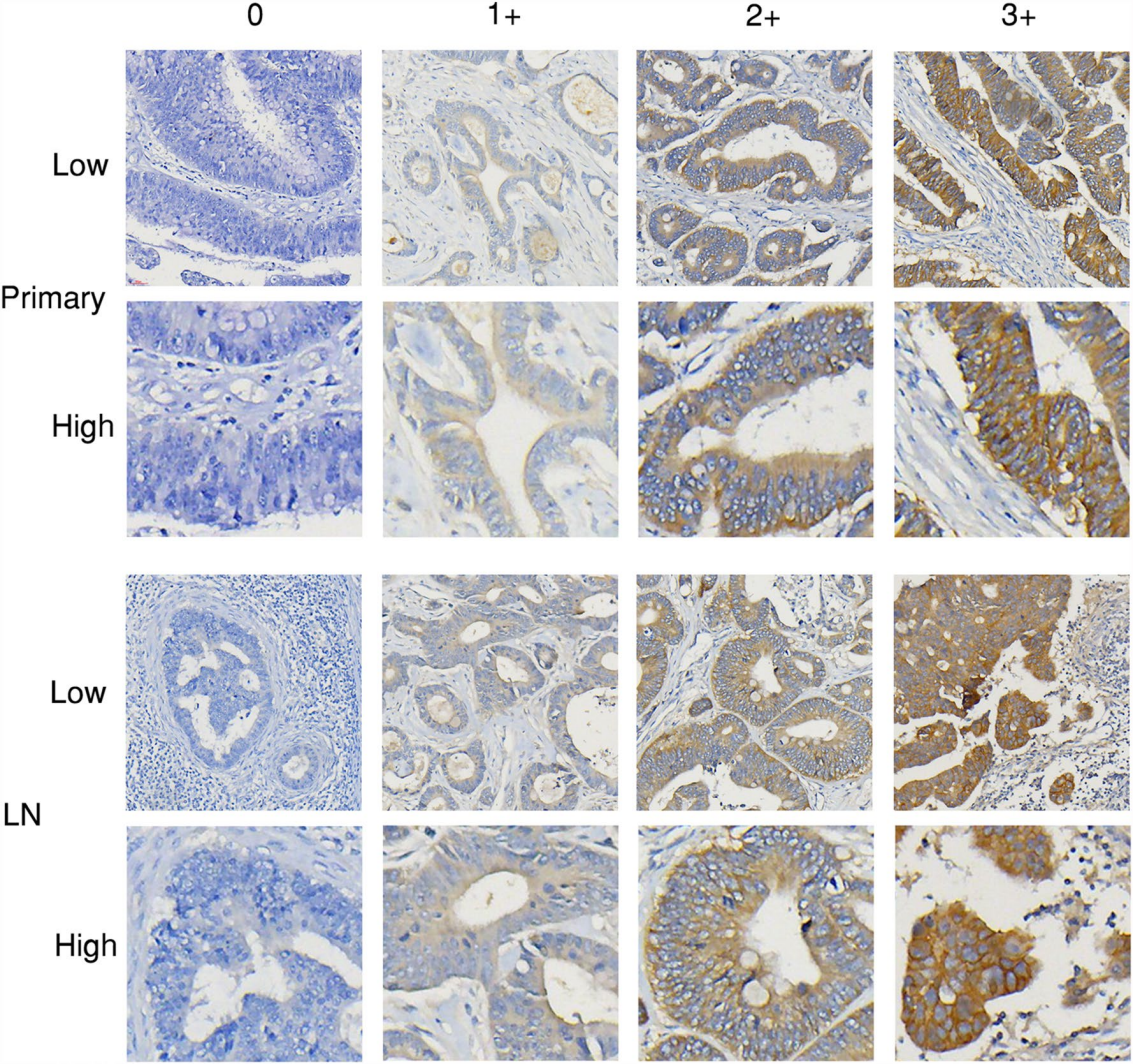
Representative images (HER2 immunohistochemistry staining). Representative images of immunohistochemistry staining scores (0, 1+, 2+, and 3+) for HER2 in primary tumor (Primary) and lymph node metastasis (LN) of CRC. Low: low magnificent power field (× 40); High: high magnificent power field (× 200).

**Figure 3:**
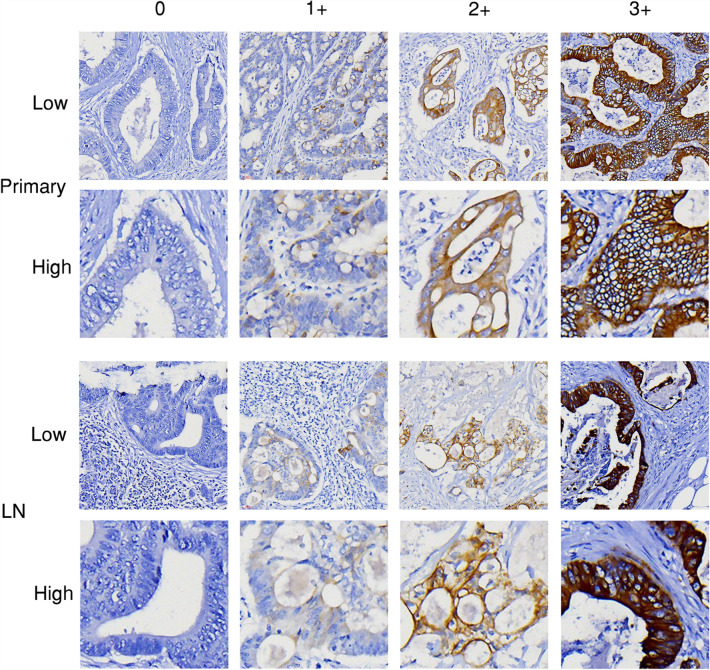
Representative images (HER3 immunohistochemistry staining). Representative images of immunohistochemistry staining scores (0, 1+, 2+, and 3+) for HER3 in primary tumor (Primary) and lymph node metastasis (LN) of CRC. Low: low magnificent power field (× 40); High: high magnificent power field (× 200).

Lymph node metastases are tumor lesions in lymph nodes located in the lymphatic drainage of primary tumor lesions. Overall, lymph node metastases showed relatively lower percentage of high EGFR, HER2, and HER3 expression (immunohistochemistry 2+/3+), compared to primary lesions. In the 79 patients, majority (52, 65.8%) of the primary lesions showed high EGFR expression (2+/3+), compared to 46 (58.2%) in lymph node metastases. High HER2 expression (2+/3+) was found in 52 (65.8%) primary lesions and 33 (41.8%) lymph node metastases. High HER3 expression (2+/3+) was found in 66 (83.5%) of primary lesions and 56 (70.9%) of lymph node metastases. Representative images of EGFR, HER2, and HER3 immunohistochemistry staining are shown in Figs. 1, 2, and 3, respectively.

### Correlation of EGFR, HER2, and HER3 scores between primary lesions and lymph node metastases

Table 2 shows a detailed comparison of EGFR, HER2, and HER3 immunohistochemistry scores between primary and metastatic lesions. As shown in Table 2, the correlation rates between primary lesion and lymph node metastases were only 67.1% (53/79) for EGFR, 63.3% (50/79) for HER2, and 74.7% (59/79) for HER3. After converting the scores into high expression (2+/3+) or low expression (0/1+), results showed higher correlation rates: 79.7% (63/79) for EGFR, 73.4% (58/79) for HER2, and 82.3% (65/79) for HER3. In the discordant samples, majority had low expression in lymph node metastases (and high expression in primary lesions): EGFR, 18/26 (69.2%); HER2, 28/29 (96.6%); HER3, 16/20 (80.0%).

**Table 2:** EGFR, HER2, and HER3 immunohistochemistry scores of primary lesions and lymph node metastases of CRC.

	Lymph node metastases														
	EGFR (n = 79)					HER2 (n = 79)					HER3 (n = 79)				
		0	1+	2+	3+		0	1+	2+	3+		0	1+	2+	3+
Primary lesions	0	8	2	0	0	0	8	0	0	0	0	7	2	1	0
	1+	3	9	4	1	1+	5	13	1	0	1+	0	2	1	0
	2+	2	8	31	1	2+	6	13	28	0	2+	5	5	41	0
	3+	0	1	4	5	3+	1	0	3	1	3+	2	0	4	9

As shown in Table 3, stage IV tumors (M1 stage) showed higher correlation rates of HER2 immunohistochemistry scores compared to stage III tumors (M0 stage) (*P* = 0.050). No significant differences were found in the correlation rates of EGFR or HER3 scores between different subgroups of clinicopathologic characteristics, or in the correlation rates of HER2 between different subgroups of gender, age, tumor type, primary tumor location, differentiation, T stage, and N stage. Furthermore, we also looked into the data of high/low expression of EGFR, HER2, and HER3 and analyzed their relationship with patient information and clinicopathologic parameters. However, no significant difference was observed (see Supplementary Table S1 for details).

**Table 3:** Correlation between patient clinicopathologic characteristics and correlation rates of EGFR, HER2, and HER3 immunohistochemistry scores between primary lesions and lymph node metastases.

Characteristics	n (%) of correlated cases versus (vs.) n (%) of uncorrelated cases					
	EGFR	*P*	HER2	*P*	HER3	*P*
**Gender**						
Male	24 (63.2%) vs. 14 (36.8%)	0.474	28 (73.7%) vs. 10 (26.3%)	0.065	29 (76.3%) vs. 9 (23.7%)	0.748
Female	29 (70.7%) vs. 12 (29.3%)		22 (53.7%) vs. 19 (46.3%)		30 (73.2%) vs. 11 (26.8%)	
**Age**						
< 67 years	24 (60.0%) vs. 16 (40.0%)	0.174	27 (67.5%) vs. 13 (32.5%)	0.432	32 (80.0%) vs. 8 (20.0%)	0.271
> 67 years	29 (74.4%) vs. 10 (25.6%)		23 (59.0%) vs. 16 (41.0%)		27 (69.2%) vs. 12 (30.8%)	
**Tumor type**						
Typical	48 (66.7%) vs. 24 (33.3%)	1.000	44 (61.1%) vs. 28 (38.9%)	0.252	53 (73.6%) vs. 19 (26.4%)	0.672
Mucinous	5 (71.4%) vs. 2 (28.6%)		6 (85.7%) vs. 1 (14.3%)		6 (85.7%) vs. 1 (14.3%)	
**Primary tumor location**						
Colon	20 (66.7%) vs. 10 (33.3%)	0.950	22 (73.3%) vs. 8 (26.7%)	0.147	24 (80.0%) vs. 6 (20.0%)	0.395
Rectum	33 (67.3%) vs. 16 (32.7%)		28 (57.1%) vs. 21 (42.9%)		35 (71.4%) vs. 14 (28.6%)	
**Differentiation**						
Low	11 (78.6%) vs. 3 (21.4%)	0.367	11 (78.6%) vs. 3 (21.4%)	0.191	11 (78.6%) vs. 3 (21.4%)	1.000
Moderate/High	42 (64.6%) vs. 23 (35.4%)		39 (60.0%) vs. 26 (40.0%)		48 (73.8%) vs. 17 (26.2%)	
**Tumor stage**						
III	37 (63.8%) vs. 21 (36.2%)	0.300	33 (56.9%) vs. 25 (43.1%)	0.050	43 (74.1%) vs. 15 (25.9%)	0.853
IV	16 (76.2%) vs. 5 (23.8%)		17 (81.0%) vs. 4 (19.0%)		16 (76.2%) vs. 5 (23.8%)	
**T stage**						
T1 and T2	3 (50.0%) vs. 3 (50.0%)	0.389	5 (83.3%) vs. 1 (16.7%)	0.406	6 (100.0%) vs. 0 (0%)	0.329
T3 and T4	50 (68.5%) vs. 23 (31.5%)		45 (61.6%) vs. 28 (38.4%)		53 (72.6%) vs. 20 (27.4%)	
**N stage**						
N1	26 (63.4%) vs. 15 (36.6%)	0.470	23 (56.1%) vs. 18 (43.9%)	0.168	27 (65.9%) vs. 14 (34.1%)	0.061
N2 and N3	27 (71.1%) vs. 11 (28.9%)		27 (71.1%) vs. 11 (28.9%)		32 (84.2%) vs. 6 (15.8%)	
**M stage**						
M0	37 (63.8%) vs. 21 (36.2%)	0.300	33 (56.9%) vs. 25 (43.1%)	0.050	43 (74.1%) vs. 15 (25.9%)	0.853
M1	16 (76.2%) vs. 5 (23.8%)		17 (81.0%) vs. 4 (19.0%)		16 (76.2%) vs. 5 (23.8%)	

A TNM classification system was used to define the tumor stage, T stage, N stage and M stage of the tumors.

## Discussion

The HER family members, EGFR, HER2, and HER3, are key treatment targets in mCRC, and anti-HER therapies are currently in clinical trials or already used in clinical practice^5,7,10^. In our study, we investigated the protein expression of those factors in paired primary lesions and lymph node metastases. Results showed moderate to high percentages of high EGFR, HER2, and HER3 protein expression (immunohistochemistry 2+ and 3+), ranging from 65.8% to 83.5% in primary lesions and 41.8% to 70.9% in lymph node metastases. However, the percentages of positive EGFR, HER2, and HER3 expression in CRC varied greatly in previous studies^22,24–26^. As summarized by Wei^22^, percentages of positive EGFR, HER2, and HER3 expression in CRC ranged from 20 to 95%, 3% to 82%, and 36% to 89%, respectively in different studies. The great variations were possibly caused by differences in patient population, antibody, and scoring criteria. For example, many of the studies used the percentage of positive-stained cells by immunohistochemistry to define positivity/negativity of HER family members^17,27–31^. Therefore, we limit our discussion within studies using similar scoring criteria to our study (4-tiered scoring criteria based on staining intensity).

Regarding EGFR expression, study by Wei^22^ reported high EGFR expression (2+ and 3+) in 29% of primary lesions and 14% of lymph node metastases. In the study by Yarom^16^, high EGFR expression was observed in 39.7% of primary tumors. In our study, high EGFR expression (2+ and 3+) was more commonly seen: 65.8% in primary tumor and 58.2% in lymph node metastases. Regarding HER2 expression, high HER2 expression was only found in 1.8% (1/55) of both primary and metastatic lesions in Wei’s study^22^. Using a slightly stricter criteria (2+ in > 50% tumor cells, instead of 10%), Shan^19^ found high HER2 expression in 11.6% of primary tumors and 10.1% of lymph node metastases. In addition, studies by Li JL^18^, Styczen^26^, Drecoll^32^, Fotiades^33^, Farzand^34^, Kavanagh^35^, Li Q^36^, Ramieri^37^, Schuell^38^, Seo^39^, Gao^40^, and Stahler^41^ reported high HER2 expression in 54.5%, 63.6%, 14%, 33.3%, 54.8%, 10%, 15.5%, 10%, 4%, 6%, 50%, and 14.4% of primary tumors of CRC, respectively. Our results showed high HER2 expression in 65.8% of primary lesions and 41.8% of lymph node metastases, which are higher than the previous studies. For HER3 expression, two studies by Ledel^12,23^ reported high HER3 expression in 80% and 70% of primary lesions, and 81% and 75% of lymph node metastases, respectively. Study by Styczen^26^ found high HER3 expression in 72.7% of primary tumors. Studies by Stahler^41^, Seo^39^, and Ledel^42^ reported high HER3 expression in 67%, 69%, and 67% of primary tumors, respectively, and our study showed high HER3 expression in 83.5% of primary tumors and 70.9% of metastases. In all, although the percentages of high HER3 expression were relatively close between previous studies and our findings, the percentages of high EGFR and HER2 expression varied greatly even when similar scoring criteria were used. This could possibly be explained by the different antibodies and experimental conditions used in those studies, highlighting the importance of standardization of reagents and experimental conditions in this field.

Our results also showed lower percentages of high EGFR, HER2, and HER3 expression in lymph node metastases compared to primary tumors (EGFR: 58.2% vs. 65.8%; HER2: 41.8% vs. 65.8%; HER3: 70.9% vs. 83.5%), which was also observed in the EGFR and HER2 expression results reported in previous studies^16,18–20,22,26^. However, unlike our results, high HER3 expression was more commonly seen in metastases rather than primary tumors in previous reports^12,23,26^.

Tumor heterogeneity is a key challenge for cancer medicine. Although targeted therapies often lead to robust initial responses, it is almost inevitable for the tumors to develop resistance and relapse, which is partially caused by intra-tumor and temporal heterogeneity (evolution of tumor cells under the pressure of treatment)^14,15,43–45^. The discrepancies in the phenotypes of tumor cells observed between primary and metastatic lesions also reflect both spatial (inter-tumor) and temporal heterogeneity^15^. In our study, we also investigated the heterogeneity of EGFR, HER2, and HER3 expression between primary lesions and lymph node metastases. Results showed moderate correlation rates in the scores of EGFR (67.1%), HER2 (63.3%), and HER3 (74.7%). For EGFR, Wei^22^ reported a 69.6% correlation rate of EGFR scores between primary tumors and lymph node metastases, which is close to our findings (67.1%). For HER2 scores, Wei^22^ reported a correlation rate of 96.4%. Shan^19^ investigated the correlation of HER2 positive (immunohistochemistry 2+/3+) and negative (0/1+) between primary tumors and lymph node metastases, and reported a correlation rate of 89.2%. Since the study populations of Wei^22^, Shan^19^, and our study were all Chinese population and our study used the same scoring criteria as the previous study by Wei^22^, the higher correlation rates found in previous studies by Wei^22^ and Shan^19^ may be partially related to the small number of HER2 high expression cases in these studies (1 and 8, respectively) which could potentially lead to bias. For HER3, in the studies by Wei^22^ and Ledel^23^, correlation of high expression (2+/3+)/low expression (0/1+) of HER3 was investigated, and results showed correlation rates of 83.7% and 82.4%, respectively, which were similar to our findings (82.3%). In all, unlike distant metastasis which showed great variations in correlation rates (EGFR: 46.5% to 94.7%^16–18^; HER2: 55% to 72.7%^18–20^), good agreement was observed in the correlation rates of EGFR expression (67.1% to 69.6%) and HER3 expression (82.3% to 83.7%) between primary tumors and lymph node metastases. Compared to our study results (63.3%), previous studies found much higher correlation rates of HER2 expression (96.4% and 89.2%), which might be due to the bias caused by small numbers of HER2 high expression cases in these studies^19,22^. Investigations in large patient cohorts are required to determine the correlation rate of HER2 expression between primary tumors and lymph node metastases. Furthermore, the significant proportions of uncorrelated EGFR, HER2, and HER3 expression levels between primary tumors and lymph node metastases indicate that it may not be appropriate to use genetic profiles of lymph node metastasis to guide treatment plans, given that genetic profiles of primary tumor are more widely-used to guide patient selection of targeted therapies in clinical trials^46–50^. Several possible methods could be used to minimize the proportion of uncorrelated cases, e.g. standardization of antibodies, experimental conditions and scoring criteria, and investigations based on large patient cohorts. However, due to the intrinsic differences in the EGFR, HER2, and HER3 expression levels between primary tumors and lymph node metastases, uncorrelated cases may still exist even after applying these methods.

In hope of finding possible relationships between clinicopathologic characteristics and correlation of EGFR, HER2, and HER3 expression, we further analyzed the number of correlated cases in different patient subgroups. Interestingly, significantly higher correlation of HER2 scores were found in stage IV tumors (M1 stage) compared to stage III tumors (M0 stage), although no significant differences were found between the correlation of clinicopathologic characteristics and EGFR or HER3 expression, or between HER2 expression and clinicopathologic characteristics other than tumor stage and M stage. To the best of our knowledge, our study is the first to analyze the relationship between clinicopathologic characteristics and the correlation of EGFR, HER2, and HER3 expression between primary and lymph node metastatic lesions of mCRC. More investigations are required to find more possible markers to predict the correlation of these factors.

Although majority of the studies used immunohistochemistry to evaluate the expression of EGFR, HER2, and HER3 in CRC, some previous investigations used other technologies, e.g. fluorescence in situ hybridization (FISH), next-generation sequencing (NGS), silver in situ hybridization (SISH), reverse transcription-polymerase chain reaction (RT-PCR), SNP-array, and microarray. In an early study by Ooi^51^, HER2 overexpression measured by immunohistochemistry showed an 100% correlation with HER2 amplification detected by FISH. In addition, HER2 overexpression in primary tumors also showed an 100% correlation with the HER2 expression of tumor cells in lymph node metastasis and/or liver metastasis^51^. However, only 25% of the paired lymph node metastases showed EGFR-overexpressing cancer cells in the 12 cases with EGFR-overexpressing primary CRC tumors^51^. In a previous study by Molinari^29^, EGFR amplification or Chromosome 7 polysomy was detected using FISH, and results showed an 84.6% (11/13) correlation rate between primary tumors and lymph node metastases. Shimada^52^ performed comprehensive genomic sequencing by NGS to detect HER2 amplification in mCRC, and the results showed a strong correlation (an 100% correlation rate) with the HER2 status determined by immunohistochemistry and FISH. Similarly, in two previous studies by Gong^53^ and EI-Deiry^54^, HER2 amplification was identified using NGS in patients with CRC, and the reported positive rates were 5.1% (7/138) and 5.7% (94/1653), respectively. In another study, HER2 amplification was measured by SISH, and results showed an 8.4% (16/191) positive rate of HER2 amplification in advanced CRC^55^. Yen^31^ used RT-PCR to measure EGFR mRNA levels, and EGFR mRNA overexpression was found in 78.9% (75/95) of CRC tumors, which was higher than the EGFR protein overexpression rate (61.1%, 58/95) measured by immunohistochemistry. In another study using RT-PCR to measure EGFR mRNA expression, a significant correlation was observed in EGFR mRNA expression levels between primary tumor and liver metastasis^56^. Del Carmen^28^ investigated EGFR expression in sporadic CRC tumors and found a strong correlation between EGFR expression measured by immunohistochemistry and EGFR gene copy number determined by FISH, SNP-array, and microarray.

In summary, our study found moderate proportions of high EGFR, HER2, and HER3 expression in primary tumors and lymph node metastases of patients with mCRC. Further analysis showed moderate correlations of the scores of EGFR, HER2, and HER3 between primary and metastatic lesions. In addition, our study for the first time showed that tumor stage and M stage could serve as predictive markers for the correlation of HER2 scores between primary tumors and lymph node metastases of mCRC. More studies with larger sample size are required to further validate those findings.

## Methods

### Patient samples

Paired primary tumor and lymph node metastasis samples were retrospectively collected from archived formalin-fixed and paraffin embedded (FFPE) samples of patients who were treatment-naïve and underwent surgery in Sichuan Provincial People's Hospital from January 2019 to June 2019. All the lymph node metastasis samples were from lymph nodes within the lymphatic drainage of the primary tumor, which were collected together with the primary tumor samples during the surgery. The histological type of the tumor was determined after reviewing by two pathologists. Clinicopathologic characteristics were also collected. The pathological TNM staging of the tumors was based on the guideline developed by Japanese Society for Cancer of the Colon and Rectum^57^. Subsequent immunohistochemistry staining was conducted in November 2021. This study was approved by the Medical Ethics Committee of Sichuan Academy of Medical Sciences and Sichuan Provincial People’s Hospital (#484, Year 2021) and in compliance with Declaration of Helsinki. Informed consent was waived by the Medical Ethics Committee since all the archival samples were retrospectively collected and all the data were collected and analyzed anonymously.

### Immunohistochemistry staining

The paired primary tumor and lymph node metastasis samples were sectioned at 4 μm and placed on the same slide. After de-paraffinization, hydration, and antigen retrieval using boiling, slides were stained using primary antibody against EGFR (RMA-0804, MXB Biotechnologies), HER2 (ab16662, Abcam), or HER3 (ab93739, Abcam). After staining with secondary antibody (ab6721, Abcam), slides were counterstained with hematoxylin and visualized using 3,3′-diaminobenzidine (DAB). Tumor samples which were stained positive in previous tests were used as positive control, and negative controls were prepared by replacing primary antibody with phosphate-buffered saline.

### Scoring criteria

The immunohistochemistry staining results were then independently scored by two pathologists (J.X. and F.L.) using pre-defined scoring criteria which were based on the 4-tiered scoring system suggested by Hofmann^58^ and criteria used for EGFR, HER2, and HER3 scoring in previous studies^12,22^. Detailed criteria are listed as follows: 0, completely negative staining or faint staining < 10% of tumor cells; 1+, faint staining of tumor cell membranes (or very faint staining in only part of the membrane for HER3); 2+, moderate staining of entire cell membrane (or in entire or basolateral membrane for HER3); 3+, strong staining of entire cell membrane (or in entire or basolateral membrane for HER3). High expression of the biomarkers was defined as immunohistochemistry scores of 2+ or 3+, while low expression was defined as immunohistochemistry scores of 0 or 1+. The pathologists were blinded to the patient characteristics information, and slides with insufficient tumor tissue in either primary lesion or lymph node metastasis were excluded in the scoring step.

### Statistical analysis

Statistical analysis was performed using SPSS 19.0 statistical software (IBM Corporation, USA). The χ^2^ test was used to analyze possible relationships between the immunohistochemistry score (or high/low expression of biomarkers) and patient clinicopathologic characteristics. *P* < 0.05 was considered statistically significant.

## Supplementary Information

Supplementary Information 1.

Supplementary Information 2.

## Data Availability

All the data were included in the article or Supplementary Information.
