# Supplementary material for: EGFR, HER2, and HER3 protein expression in paired primary tumor and lymph node metastasis of colorectal cancer
Source: Sci Rep. 2022 Jul 28;12:12894. doi: 10.1038/s41598-022-17210-2 (PMC9334602; doi:10.1038/s41598-022-17210-2)
Supplement: Supplementary file 1 — Supplementary Information 1. [file 41598_2022_17210_MOESM1_ESM.docx]

**Supplementary Table S1. Correlation between patient clinicopathologic characteristics and correlation rates of high/low expression of EGFR, HER2, and HER3 between primary lesions and lymph node metastases.**

| Characteristics | n (%) of correlated cases versus (vs.) n (%) of uncorrelated cases | | | | | |
| --- | --- | --- | --- | --- | --- | --- |
|  | EGFR (n = 79) | | HER2 (n = 79) | | HER3 (n = 79) | |
| Gender | | | | | | |
| Male | 31 (81.6%) vs. 7 (18.4%) | *P* = 0.696 | 31 (81.6%) vs. 7 (18.4%) | *P* = 0.114 | 30 (78.9%) vs. 8 (21.1%) | *P* = 0.455 |
| Female | 32 (78.0%) vs. 9 (22.0%) |  | 27 (65.9%) vs. 14 (34.1%) |  | 35 (85.4%) vs. 6 (14.6%) |  |
| Age | | | | | | |
| < 67 years | 30 (75.0%) vs. 10 (25.0%) | *P* = 0.288 | 29 (72.5%) vs. 11 (27.5%) | *P* = 0.852 | 34 (85.0%) vs. 6 (15.0%) | *P* = 0.521 |
| > 67 years | 33 (84.6%) vs. 6 (15.4%) |  | 29 (74.4%) vs. 10 (25.6%) |  | 31 (79.5%) vs. 8 (20.5%) |  |
| Tumor type | | | | | | |
| Typical | 57 (79.2%) vs. 15 (20.8%) | *P* = 1.000 | 51 (70.8%) vs. 21 (29.2%) | *P* = 0.180 | 59 (81.9%) vs. 13 (18.1%) | *P* = 1.000 |
| Mucinous | 6 (85.7%) vs. 1 (14.3%) |  | 7 (100.0%) vs. 0 (0%) |  | 6 (85.7%) vs. 1 14.3%) |  |
| Primary tumor location | | | | | | |
| Colon | 24 (80.0%) vs. 6 (20.0%) | *P* = 0.965 | 25 (83.3%) vs. 5 (16.7%) | *P* = 0.119 | 27 (90.0%) vs. 3 (10.0%) | *P* = 0.160 |
| Rectum | 39 (79.6%) vs. 10 (20.4%) |  | 33 (67.3%) vs. 16 (32.7%) |  | 38 (77.6%) vs. 11 (22.4%) |  |
| Differentiation | | | | | | |
| Low | 12(85.7%) vs. 2 (14.3%) | *P* = 0.723 | 13 (92.9%) vs. 1 (7.1%) | *P* = 0.097 | 13 (92.9%) vs. 1 (7.1%) | *P* = 0.444 |
| Moderate to High | 51 (78.5%) vs. 14 (21.5%) |  | 45 (69.2%) vs. 20 (30.8%) |  | 52 (80.0%) vs. 13 (20.0%) |  |
| Tumor stage | | | | | | |
| III | 45 (77.6%) vs. 13 (22.4%) | *P* = 0.538 | 40 (69.0%) vs. 18 (31.0%) | *P* = 0.137 | 47 (81.0%) vs. 11 (19.0%) | *P* = 0.749 |
| IV | 18 (85.7%) vs. 3 (14.3%) |  | 18 (85.7%) vs. 3 (14.3%) |  | 18 (85.7%) vs. 3 (14.3%) |  |
| T stage | | | | | | |
| T1 and T2 | 3 (50.0%) vs. 3 (50.0%) | *P* = 0.094 | 5 (83.3%) vs. 1 (16.7%) | *P* = 1.000 | 6 (100.0%) vs. 0 (0%) | *P* = 0.584 |
| T3 and T4 | 60 (82.2%) vs. 13 (17.8%) |  | 53 (72.6%) vs. 20 (27.4%) |  | 59 (80.8%) vs. 14 (19.2%) |  |
| N stage | | | | | | |
| N1 | 31 (75.6%) vs. 10 (24.4%) | *P* = 0.342 | 28 (68.3%) vs. 13 (31.7%) | *P* = 0.284 | 32 (78.0%) vs. 9 (22.0%) | *P* = 0.306 |
| N2 & N3 | 32 (84.2%) vs. 6 (15.8%) |  | 30 (78.9%) vs. 8 (21.1%) |  | 33 (86.8%) vs. 5 (13.2%) |  |
| M stage | | | | | | |
| M0 | 45 (77.6%) vs. 13 (22.4%) | *P* = 0.538 | 40 (69.0%) vs. 18 (31.0%) | *P* = 0.137 | 47 (81.0%) vs. 11 (19.0%) | *P* = 0.749 |
| M1 | 18 (85.7%) vs. 3 (14.3%) |  | 18 (85.7%) vs. 3 (14.3%) |  | 18 (85.7%) vs. 3 (14.3%) |  |

A TNM classification system[^57^](#_ENREF_57) was used to define the tumor stage, T stage, N stage and M stage of the tumors.
