# Supplementary material for: EGFR, HER2, and HER3 protein expression in paired primary tumor and lymph node metastasis of colorectal cancer
Source: Sci Rep. 2022 Jul 28;12:12894. doi: 10.1038/s41598-022-17210-2 (PMC9334602; doi:10.1038/s41598-022-17210-2)
Supplement: Supplementary file 2 — Supplementary Information 2. [file 41598_2022_17210_MOESM2_ESM.docx]

**Supplementary Table S2. Detailed data of patient clinicopathologic characteristics, expression of EGFR, HER2, and HER3 and their correlations between primary lesions and lymph node metastases.**

| **Patient ID** | **Age (years)** | **Gender** | **Tumor type** | **Tumor location** | **Differentiation** | **T stage** | **N stage** | **M stage** | **Tumor stage** | **EGFR (primary lesion)** | **EGFR (lymph node metastases)** | **Correlation (EGFR)** | **Correlation_high&low (EGFR)** | **HER2 (primary lesion)** | **HER2 (lymph node metastases)** | **Correlation (HER2)** | **Correlation_high&low (HER2)** | **HER3 (primary lesion)** | **HER3 (lymph node metastases)** | **Correlation (HER3** | **Correlation_high&low (HER3)** |
| --- | --- | --- | --- | --- | --- | --- | --- | --- | --- | --- | --- | --- | --- | --- | --- | --- | --- | --- | --- | --- | --- |
| 1 | 67 | Male | Typical | Right | High | T3/T4 | 1 | 0 | III | 2+ | 2+ | Yes | Yes | 2+ | 1+ | No | No | 2+ | 2+ | Yes | Yes |
| 2 | 78 | Male | Typical | Left | Moderate | T3/T4 | 2 | 1 | IV | 2+ | 2+ | Yes | Yes | 1+ | 1+ | Yes | Yes | 2+ | 2+ | Yes | Yes |
| 3 | 42 | Female | Typical | Rectum | Moderate | T3/T4 | 2 | 0 | III | 2+ | 2+ | Yes | Yes | 1+ | 2+ | No | No | 2+ | 2+ | Yes | Yes |
| 4 | 73 | Male | Typical | Rectum | Moderate | T3/T4 | 1 | 0 | III | 1+ | 0 | No | Yes | 1+ | 0 | No | Yes | 2+ | 0 | No | No |
| 5 | 54 | Female | Typical | Rectum | Moderate | T3/T4 | 1 | 0 | III | 2+ | 2+ | Yes | Yes | 2+ | 1+ | No | No | 2+ | 2+ | Yes | Yes |
| 6 | 87 | Male | Typical | Right | Low | T3/T4 | 3 | 0 | III | 2+ | 0 | No | No | 1+ | 0 | No | Yes | 2+ | 2+ | Yes | Yes |
| 7 | 74 | Male | Typical | Left | Moderate | T3/T4 | 1 | 0 | III | 2+ | 2+ | Yes | Yes | 2+ | 2+ | Yes | Yes | 2+ | 2+ | Yes | Yes |
| 8 | 64 | Male | Typical | Left | Moderate | T3/T4 | 2 | 1 | IV | 3+ | 3+ | Yes | Yes | 0 | 0 | Yes | Yes | 2+ | 2+ | Yes | Yes |
| 9 | 64 | Female | Typical | Rectum | Low | T3/T4 | 2 | 1 | IV | 3+ | 3+ | Yes | Yes | 2+ | 2+ | Yes | Yes | 2+ | 2+ | Yes | Yes |
| 10 | 55 | Male | Mucinous | Left | Low | T3/T4 | 2 | 0 | III | 3+ | 3+ | Yes | Yes | 0 | 0 | Yes | Yes | 2+ | 2+ | Yes | Yes |
| 11 | 61 | Female | Typical | Rectum | Moderate | T1/T2 | 2 | 0 | III | 2+ | 2+ | Yes | Yes | 0 | 0 | Yes | Yes | 2+ | 2+ | Yes | Yes |
| 12 | 81 | Female | Typical | Rectum | Moderate | T3/T4 | 2b | 0 | III | 2+ | 0 | No | No | 2+ | 0 | No | No | 3+ | 0 | No | No |
| 13 | 62 | Female | Mucinous | Left | Low | T3/T4 | 1 | 1 | IV | 1+ | 2+ | No | No | 0 | 0 | Yes | Yes | 3+ | 3+ | Yes | Yes |
| 14 | 60 | Female | Mucinous | Rectum | Low | T3/T4 | 1 | 0 | III | 1+ | 1+ | Yes | Yes | 1+ | 0 | No | Yes | 2+ | 0 | No | No |
| 15 | 62 | Female | Typical | Rectum | High | T1/T2 | 1 | 0 | III | 3+ | 1+ | No | No | 2+ | 1+ | No | No | 2+ | 2+ | Yes | Yes |
| 16 | 85 | Female | Typical | Right | Moderate | T3/T4 | 2 | 0 | III | 0 | 0 | Yes | Yes | 2+ | 2+ | Yes | Yes | 3+ | 3+ | Yes | Yes |
| 17 | 57 | Male | Typical | Rectum | Low | T3/T4 | 2b | 0 | III | 1+ | 1+ | Yes | Yes | 3+ | 3+ | Yes | Yes | 2+ | 2+ | Yes | Yes |
| 18 | 69 | Male | Typical | Right | Moderate | T3/T4 | 2b | 1 | IV | 2+ | 2+ | Yes | Yes | 2+ | 1+ | No | No | 3+ | 3+ | Yes | Yes |
| 19 | 74 | Male | Typical | Left | Moderate | T3/T4 | 1 | 0 | III | 2+ | 1+ | No | No | 2+ | 2+ | Yes | Yes | 3+ | 3+ | Yes | Yes |
| 20 | 80 | Female | Typical | Rectum | Moderate | T3/T4 | 1 | 0 | III | 2+ | 2+ | Yes | Yes | 2+ | 1+ | No | No | 3+ | 2+ | No | Yes |
| 21 | 66 | Female | Mucinous | Right | Low | T3/T4 | 2 | 0 | III | 0 | 0 | Yes | Yes | 0 | 0 | Yes | Yes | 0 | 0 | Yes | Yes |
| 22 | 66 | Female | Typical | Rectum | Moderate | T3/T4 | 2a | 0 | III | 1+ | 2+ | No | No | 2+ | 0 | No | No | 3+ | 3+ | Yes | Yes |
| 23 | 78 | Male | Typical | Rectum | Low | T3/T4 | 1 | 1 | IV | 1+ | 1+ | Yes | Yes | 2+ | 0 | No | No | 0 | 0 | Yes | Yes |
| 24 | 51 | Male | Typical | Rectum | Low | T3/T4 | 3 | 0 | III | 0 | 0 | Yes | Yes | 2+ | 2+ | Yes | Yes | 0 | 0 | Yes | Yes |
| 25 | 63 | Female | Typical | Rectum | Low | T3/T4 | 1 | 0 | III | 3+ | 3+ | Yes | Yes | 2+ | 2+ | Yes | Yes | 0 | 1+ | No | Yes |
| 26 | 86 | Female | Typical | Rectum | Moderate | T3/T4 | 2 | 0 | III | 2+ | 2+ | Yes | Yes | 2+ | 1+ | No | No | 2+ | 2+ | Yes | Yes |
| 27 | 82 | Female | Typical | Rectum | Moderate | T3/T4 | 2 | 0 | III | 2+ | 2+ | Yes | Yes | 2+ | 1+ | No | No | 3+ | 3+ | Yes | Yes |
| 28 | 42 | Male | Typical | Rectum | Moderate | T3/T4 | 1 | 1 | IV | 2+ | 1+ | No | No | 2+ | 2+ | Yes | Yes | 2+ | 2+ | Yes | Yes |
| 29 | 83 | Male | Typical | Right | Low | T3/T4 | 2 | 1 | IV | 1+ | 1+ | Yes | Yes | 1+ | 1+ | Yes | Yes | 0 | 1+ | No | Yes |
| 30 | 53 | Female | Typical | Rectum | Moderate | T3/T4 | 2a | 1 | IV | 1+ | 1+ | Yes | Yes | 1+ | 1+ | Yes | Yes | 2+ | 2+ | Yes | Yes |
| 31 | 86 | Female | Typical | Left | Moderate | T3/T4 | 2a | 0 | III | 0 | 0 | Yes | Yes | 1+ | 1+ | Yes | Yes | 2+ | 2+ | Yes | Yes |
| 32 | 66 | Male | Typical | Rectum | Moderate | T3/T4 | 1 | 0 | III | 1+ | 2+ | No | No | 2+ | 0 | No | No | 2+ | 2+ | Yes | Yes |
| 33 | 82 | Female | Typical | Rectum | Moderate | T3/T4 | 1 | 1 | IV | 0 | 0 | Yes | Yes | 2+ | 2+ | Yes | Yes | 2+ | 2+ | Yes | Yes |
| 34 | 60 | Female | Typical | Left | Moderate | T1/T2 | 1 | 0 | III | 1+ | 2+ | No | No | 0 | 0 | Yes | Yes | 2+ | 2+ | Yes | Yes |
| 35 | 75 | Male | Typical | Rectum | Moderate | T3/T4 | 2 | 1 | IV | 2+ | 2+ | Yes | Yes | 2+ | 2+ | Yes | Yes | 2+ | 2+ | Yes | Yes |
| 36 | 70 | Female | Typical | Rectum | High | T3/T4 | 1 | 0 | III | 1+ | 1+ | Yes | Yes | 1+ | 0 | No | Yes | 1+ | 1+ | Yes | Yes |
| 37 | 52 | Female | Typical | Rectum | Moderate | T3/T4 | 1 | 0 | III | 1+ | 0 | No | Yes | 2+ | 0 | No | No | 2+ | 0 | No | No |
| 38 | 64 | Female | Typical | Rectum | Moderate | T3/T4 | 1 | 0 | III | 0 | 0 | Yes | Yes | 2+ | 1+ | No | No | 2+ | 2+ | Yes | Yes |
| 39 | 71 | Male | Typical | Left | Moderate | T3/T4 | 1 | 0 | III | 2+ | 1+ | No | No | 2+ | 2+ | Yes | Yes | 2+ | 2+ | Yes | Yes |
| 40 | 80 | Female | Typical | Right | Moderate | T3/T4 | 3 | 1 | IV | 2+ | 2+ | Yes | Yes | 1+ | 1+ | Yes | Yes | 0 | 0 | Yes | Yes |
| 41 | 46 | Female | Typical | Left | Moderate | T3/T4 | 1 | 1 | IV | 2+ | 2+ | Yes | Yes | 2+ | 1+ | No | No | 3+ | 2+ | No | Yes |
| 42 | 72 | Male | Mucinous | Rectum | Low | T3/T4 | 2b | 0 | III | 0 | 1+ | No | Yes | 0 | 0 | Yes | Yes | 2+ | 2+ | Yes | Yes |
| 43 | 65 | Female | Typical | Rectum | Moderate | T3/T4 | 2 | 0 | III | 2+ | 2+ | Yes | Yes | 1+ | 1+ | Yes | Yes | 0 | 0 | Yes | Yes |
| 44 | 77 | Female | Typical | Right | High | T3/T4 | 2b | 0 | III | 3+ | 3+ | Yes | Yes | 3+ | 2+ | No | Yes | 3+ | 3+ | Yes | Yes |
| 45 | 68 | Male | Typical | Rectum | Moderate | T3/T4 | 2 | 0 | III | 2+ | 2+ | Yes | Yes | 2+ | 2+ | Yes | Yes | 2+ | 2+ | Yes | Yes |
| 46 | 65 | Male | Typical | Left | Moderate | T3/T4 | 1 | 1 | IV | 1+ | 3+ | No | No | 1+ | 1+ | Yes | Yes | 2+ | 2+ | Yes | Yes |
| 47 | 50 | Male | Typical | Rectum | High | T3/T4 | 1 | 0 | III | 2+ | 2+ | Yes | Yes | 2+ | 2+ | Yes | Yes | 3+ | 3+ | Yes | Yes |
| 48 | 75 | Female | Typical | Rectum | Moderate | T3/T4 | 1 | 0 | III | 2+ | 2+ | Yes | Yes | 2+ | 1+ | No | No | 2+ | 0 | No | No |
| 49 | 61 | Female | Typical | Right | Moderate | T3/T4 | 1 | 0 | III | 3+ | 2+ | No | Yes | 2+ | 2+ | Yes | Yes | 2+ | 2+ | Yes | Yes |
| 50 | 65 | Male | Typical | Rectum | Moderate | T3/T4 | 2b | 0 | III | 2+ | 3+ | No | Yes | 1+ | 1+ | Yes | Yes | 2+ | 2+ | Yes | Yes |
| 51 | 75 | Male | Typical | Rectum | Moderate | T3/T4 | 2 | 0 | III | 2+ | 2+ | Yes | Yes | 2+ | 2+ | Yes | Yes | 2+ | 0 | No | No |
| 52 | 53 | Female | Typical | Left | Moderate | T3/T4 | 2 | 0 | III | 2+ | 2+ | Yes | Yes | 2+ | 1+ | No | No | 2+ | 2+ | Yes | Yes |
| 53 | 54 | Male | Typical | Right | Moderate | T3/T4 | 1 | 1 | IV | 2+ | 2+ | Yes | Yes | 1+ | 1+ | Yes | Yes | 2+ | 1+ | No | No |
| 54 | 56 | Female | Typical | Rectum | Moderate | T3/T4 | 1 | 0 | III | 2+ | 2+ | Yes | Yes | 1+ | 1+ | Yes | Yes | 0 | 0 | Yes | Yes |
| 55 | 74 | Male | Typical | Left | Moderate | T3/T4 | 2 | 1 | IV | 1+ | 1+ | Yes | Yes | 2+ | 2+ | Yes | Yes | 2+ | 2+ | Yes | Yes |
| 56 | 71 | Male | Typical | Rectum | Moderate | T3/T4 | 2 | 0 | III | 2+ | 2+ | Yes | Yes | 2+ | 2+ | Yes | Yes | 3+ | 3+ | Yes | Yes |
| 57 | 55 | Female | Typical | Rectum | Moderate | T3/T4 | 1 | 0 | III | 1+ | 1+ | Yes | Yes | 2+ | 2+ | Yes | Yes | 1+ | 2+ | No | No |
| 58 | 66 | Male | Typical | Rectum | Moderate | T3/T4 | 1 | 0 | III | 2+ | 1+ | No | No | 2+ | 2+ | Yes | Yes | 2+ | 2+ | Yes | Yes |
| 59 | 71 | Male | Typical | Left | Moderate | T3/T4 | 1 | 0 | III | 3+ | 2+ | No | Yes | 2+ | 1+ | No | No | 2+ | 1+ | No | No |
| 60 | 69 | Male | Typical | Rectum | Moderate | T3/T4 | 1 | 0 | III | 2+ | 2+ | Yes | Yes | 1+ | 0 | No | Yes | 2+ | 2+ | Yes | Yes |
| 61 | 56 | Male | Typical | Rectum | Moderate | T3/T4 | 1 | 0 | III | 2+ | 2+ | Yes | Yes | 2+ | 0 | No | No | 2+ | 2+ | Yes | Yes |
| 62 | 54 | Male | Typical | Rectum | Moderate | T3/T4 | 2 | 0 | III | 3+ | 2+ | No | Yes | 2+ | 2+ | Yes | Yes | 0 | 2+ | No | No |
| 63 | 55 | Female | Typical | Right | Moderate | T3/T4 | 1 | 1 | IV | 2+ | 2+ | Yes | Yes | 1+ | 1+ | Yes | Yes | 2+ | 2+ | Yes | Yes |
| 64 | 78 | Female | Typical | Rectum | Moderate | T3/T4 | 1b | 1 | IV | 0 | 0 | Yes | Yes | 2+ | 2+ | Yes | Yes | 2+ | 1+ | No | No |
| 65 | 64 | Male | Typical | Left | High | T3/T4 | 1 | 1 | IV | 3+ | 2+ | No | Yes | 2+ | 2+ | Yes | Yes | 2+ | 1+ | No | No |
| 66 | 62 | Female | Typical | Rectum | Moderate | T3/T4 | 1 | 0 | III | 2+ | 2+ | Yes | Yes | 2+ | 1+ | No | No | 2+ | 2+ | Yes | Yes |
| 67 | 54 | Female | Mucinous | Right | Low | T3/T4 | 1 | 1 | IV | 2+ | 2+ | Yes | Yes | 2+ | 2+ | Yes | Yes | 2+ | 2+ | Yes | Yes |
| 68 | 83 | Male | Typical | Rectum | Moderate | T3/T4 | 2a | 0 | III | 1+ | 0 | No | Yes | 3+ | 0 | No | No | 3+ | 0 | No | No |
| 69 | 86 | Female | Typical | Rectum | Moderate | T3/T4 | 2 | 0 | III | 2+ | 1+ | No | No | 1+ | 1+ | Yes | Yes | 2+ | 2+ | Yes | Yes |
| 70 | 48 | Female | Typical | Rectum | Moderate | T3/T4 | 2a | 0 | III | 2+ | 1+ | No | No | 2+ | 2+ | Yes | Yes | 2+ | 2+ | Yes | Yes |
| 71 | 76 | Female | Typical | Left | Moderate | T3/T4 | 1 | 0 | III | 1+ | 1+ | Yes | Yes | 2+ | 2+ | Yes | Yes | 3+ | 2+ | No | Yes |
| 72 | 85 | Male | Typical | Rectum | Moderate | T1/T2 | 1 | 0 | III | 2+ | 2+ | Yes | Yes | 2+ | 2+ | Yes | Yes | 2+ | 2+ | Yes | Yes |
| 73 | 34 | Female | Typical | Rectum | Moderate | T1/T2 | 2 | 0 | III | 2+ | 1+ | No | No | 1+ | 1+ | Yes | Yes | 2+ | 2+ | Yes | Yes |
| 74 | 69 | Male | Typical | Rectum | Moderate | T1/T2 | 1 | 0 | III | 2+ | 2+ | Yes | Yes | 2+ | 2+ | Yes | Yes | 1+ | 1+ | Yes | Yes |
| 75 | 83 | Male | Typical | Rectum | Moderate | T3/T4 | 2 | 0 | III | 2+ | 2+ | Yes | Yes | 2+ | 2+ | Yes | Yes | 2+ | 1+ | No | No |
| 76 | 78 | Male | Typical | Rectum | Moderate | T3/T4 | 2 | 0 | III | 2+ | 2+ | Yes | Yes | 2+ | 2+ | Yes | Yes | 2+ | 2+ | Yes | Yes |
| 77 | 53 | Female | Typical | Right | Moderate | T3/T4 | 2b | 1 | IV | 0 | 1+ | No | Yes | 3+ | 2+ | No | Yes | 2+ | 2+ | Yes | Yes |
| 78 | 80 | Male | Mucinous | Left | Low | T3/T4 | 1 | 0 | III | 0 | 0 | Yes | Yes | 0 | 0 | Yes | Yes | 0 | 0 | Yes | Yes |
| 79 | 67 | Female | Typical | Rectum | Moderate | T3/T4 | 1b | 0 | III | 2+ | 1+ | No | No | 3+ | 2+ | No | Yes | 3+ | 2+ | No | Yes |
